# Elderly patients with concurrent hip fracture and lower respiratory tract infection: the pathogens and prognosis over different bedridden periods

**DOI:** 10.1186/s13018-021-02399-1

**Published:** 2021-04-13

**Authors:** Yuan Yuan, Wei Tian, Xiaohui Deng, Rui Yue, Xiaozhu Ge, Xinbao Wu, Ping Zhang

**Affiliations:** 1 Department of Geriatrics, Beijing Jishuitan Hospital, No.31 Xinjiekou East Street, Xicheng District, Beijing, 100035 China; 2 Department of Orthopedic Surgery, Beijing Jishuitan Hospital, Beijing, 100035 China

**Keywords:** Elderly, Hip fracture, Lower respiratory tract infection

## Abstract

**Background:**

Elderly patients who experience hip fractures often become bedridden and are at risk of developing lower respiratory tract infections. The current study was to investigate the etiology and bacterial drug resistance patterns of elderly patients with hip fractures and lower respiratory tract infections on prolonged bedridden time and to determine their prognosis.

**Methods:**

Patients diagnosed with hip fractures admitted from May 2015 to April 2017 were included. The basic characteristics including the patients’ gender, age, fracture type, operation mode, bedridden duration, length of hospital stay, prognosis, past medical history, routine bloodwork, C-reactive protein (CRP), procalcitonin (PCT), blood biochemistry, blood gas analysis, glycosylated hemoglobin (HbA1C%), sputum smear, sputum culture, and anti-infection and related therapy were recorded. All patients were classified into three groups based on bed rest duration, including short-term (<1 month), mid-term (1–12 months), and long-term (> 12 months). The correlation between the bedridden time and the patients’ basic characteristics, disease history, laboratory examination results, pathogen, anti-infection, and related therapy were evaluated. The risk factors related to the prognosis of the disease were investigated.

**Results:**

Prolonged bed rest in patients led to an increase in hospitalization time, mortality rates, and decreased serum albumin levels (*P* < 0.05). Sputum bacteriological culture results showed that, with bed rest prolongation, the proportion of *Pseudomonas aeruginosa* and fungal infections increased. Binomial logistic regression of pulmonary infection prognosis, glucocorticoid use during the anti-infective period, prolonged bedridden time, and serum albumin level showed that intravenous use of glucocorticoid during anti-infective treatment, bed rest > 1 year, and low serum albumin level were related to poor prognosis.

**Conclusion:**

Elderly hip fracture patients with prolonged bedridden time had an increased chance of opportunistic pulmonary infection and decreased nutritional status. Glucocorticoids should be used cautiously.

**Supplementary Information:**

The online version contains supplementary material available at 10.1186/s13018-021-02399-1.

## Background

With the population aging globally, hip fractures, often known as the “last fracture of life”, have become a common disease threatening the health of the elderly. The incidence of hip fracture in the elderly was 1.7 million in 1990 and is estimated to reach 6.3 million in 2050 [1]. Approximately two thirds of elderly patients with hip fractures lost their self-care ability in their daily life [2]. One quarter of elderly patients die of hip fracture associated complications within 1 year [2–5].

About 0.3–3.2% of elderly patients with acute hip fractures have a lower respiratory tract infection before admission [6, 7]. The incidence of lower respiratory tract infections rises to 13.9% within 30 days after surgery [8]. In addition, approximately 6.9–17% of elderly patients with hip fractures died within 30 days after surgery [9, 10]. The extent to which a lower respiratory tract infection would affect the development of disease in elderly patients with hip fractures has been investigated. Several studies have shown that lower respiratory tract infection is not only the primary cause of perioperative death in elderly patients with hip fractures but also the primary cause of 30 days re-admission after surgery and the main cause of high mortality at 30 days after surgery [7, 11–13]. It has been shown that the high mortality of hip fracture lasts for 1 year after injury [7, 13], while another study has found that the high mortality may continue for more than 20 years after injury [14].

At present, there were few studies on the causative pathogens of lung infections over different bedridden periods. Therefore, it is vital to conduct an in-depth study on the composite disease state and the diagnosis and treatment of hip fractures and lower respiratory tract infections in the elderly. This study was designed to investigate the etiology and bacterial drug resistance patterns of elderly patients with hip fractures and lower respiratory tract infections on prolonged bedridden time and to determine their prognosis.

## Methods

### Patients

Patients with acute hip fractures and lower respiratory tract infection and the patients with a hip fracture history and subsequent disabilities, and lower respiratory tract infections admitted to our hospital from May 2015 to April 2017 were included. This retrospective study was approved by the Ethics Committee of our hospital.

The inclusion criteria were (1) age (≥ 65 years old); (2) acute hip fracture diagnosed by an imaging examination or patients had a clear history of hip fracture, which subsequently led to the limitation or loss of the patients' abilities to live independently or move freely (i.e., due to disability); (3) lower respiratory tract infection met the criterion of Chinese diagnosis and treatment of community-acquired pneumonia in adults (2016) [15], including infection of the pulmonary parenchyma (alveolar wall or pulmonary interstitium), community-acquired pneumonia, and pneumonia caused by a latent pathogenic infection in the period following admission; (4) blood tests, sputum specimens, and chest imaging examination completed within 72 h of admission; and (5) complete medical records and laboratory examinations. The exclusion criteria were hospital-acquired pneumonia (HAP), which was defined as pneumonia that developed at least 48 h following hospitalization, according to the 2016 Guidelines for the Management of adults with hospital-acquired or ventilator-associated pneumonia [11].

### Observation index

The original medical records including the patient’s gender, age, fracture type, operation mode, bedridden duration, length of hospital stay, prognosis, past medical history, routine bloodwork, C-reactive protein (CRP), procalcitonin (PCT), blood biochemistry, blood gas analysis, glycosylated hemoglobin (HbA1C%), sputum smear, sputum culture, and anti-infection and related therapy were recorded.

### Correlation analysis

The patients were divided into a short-term bedridden group (< 1 month), a medium-term bedridden group (1–12 months), and long-term bedridden group (> 12 months) according to the length of time the patient remained in bed after the hip fracture. The correlation between the bedridden time and the patients’ basic characteristics, disease history, laboratory examination results, pathogen, anti-infection, and related therapy were evaluated.

### Univariate analysis and binomial logistic regression analysis related to disease prognosis

All the collected variables were included in the univariate analysis of death-related factors. The risk factors related to the prognosis of disease included: advanced age, > 1 month bedridden time after hip fracture, long-term nasogastric feeding, multiple hospitalizations in the past year, length of hospital stay, low serum albumin level, infection by drug-resistant bacteria or by drug-resistant fungi, third-generation cephalosporins (without enzyme inhibitors), expansion of antibiotic coverage, use of antifungal drugs, use of glucocorticoids, need for parenteral nutritional support, and endotracheal intubation or tracheotomy. These single factors were further incorporated into the binomial logistic regression model to investigate the risk factors related to the prognosis of the disease.

### Statistical analysis

All the data were analyzed using the SAS statistical software version 9.4 (SAS Institute Inc. Cary, NC, USA). Measurement data were expressed by the mean and standard deviation (*x* ± *s*) or median (IQR), and counting data were expressed by rate. Non-normally distributed, continuous measurement data were analyzed with a one-way ANOVA or the Kruskal-Wallis test. Categorical counting data was assessed with the *χ*^2^ test or Fisher’s exact test. The prognostic factors were included in the single factor logistic regression analysis. The variables with *P* < 0.05 were further included in logistic regression analysis, and model selection was performed based on stepwise criteria. Odds ratios (OR) and the corresponding 95% confidence intervals were reported. *P* < 0.05 was considered a statistically significant difference.

## Results

### Basic characteristics

A total of 101 elderly bedridden patients with hip fractures and lower respiratory tract infections were enrolled in the study, including 46 cases in the short-term bedridden group (45.5%), 23 cases in the medium-term bedridden group (22.8%), and 32 cases in the long-term bedridden group (31.7%). The average age was 82.31 ± 7.98 years. The proportion of females (55 cases, 54.5%) was slightly higher than that of males (46 cases, 45.6%). The proportion of femoral neck fractures (64 cases, 63.4%) was slightly higher than that of intertrochanteric fractures (37 cases, 36.6%). Fracture management included joint replacement (48 cases, 47.5%), internal fixation (29 cases, 28.7%), and conservative treatment (24 cases, 23.8%). In total, 17 patients died, 16 of whom (94.12%) died due to severe pneumonia (Supplementary Table 1).

The correlation analysis showed that with the prolongation of bedridden time, the proportion of patients over 80 years old (*R* = 6.578, *P* = 0.037), the number of hospitalizations in the past year (*R* = 0.525, *P* < 0.001), and the number of days hospitalized (*R* = 0.515, *P* < 0.001) showed a significant upward trend. Moreover, the mortality rate also increased significantly (*R* = 0.338, *P* < 0.001).

### Medical history

Of the 101 patients, 94 (93.1%) had underlying diseases, including hypertension (68 patients, 67.3%), cerebrovascular disease (57 patients, 56.4%), anemia (51 patients, 50.5%), coronary artery disease (40 patients, 39.6%), diabetes (25 patients, 24.8%), dementia (24 patients, 24%), and arrhythmia (21 patients, 20.8%). Longer bedridden time was correlated with a number of underlying diseases, cerebrovascular disease, coronary artery disease, arrhythmia, smoking history, and indwelling urinary catheters (*P* < 0.05) (Table 1).

**Table 1:** Comparison of underlying diseases in elderly bedridden patients with hip fracture and lower respiratory tract infection

Underlying diseases	Total	Short-term bedridden group	Middle-term bedridden group	Long-term bedridden group	***P***
***n*** **(%)/M ± SD**	*n* = 101	*n* = 46	*n* = 23	*n* = 32	
**Complicated with underlying diseases**	94 (93.1%)	42 (91.3%)	22 (95.7%)	30 (93.8%)	0.785
**Number of underlying diseases**	3.06 ± 1.70	2.27 ± 1.47	3.48 ± 1.38	3.88 ± 1.76	< 0.001
**High blood pressure**	68 (67.3%)	26 (56.5%)	19 (82.6%)	23 (71.9%)	0.075
**Cerebrovascular disease**	57 (56.4%)	14 (30.4%)	21 (91.3%)	22 (68.8%)	< 0.001
**Anemia**	51 (50.5%)	23 (50%)	12 (52.2%)	16 (50%)	0.983
**Coronary heart disease**	40 (39.6%)	9 (19.6%)	13 (56.5%)	18 (56.3%)	< 0.001
**Diabetes**	25 (24.8%)	9 (19.6%)	9 (39.1%)	7 (21.9%)	0.168
**Alzheimer’s disease**	24 (24.0%)	7 (15.6%)	4 (17.4%)	13 (40.6%)	0.035
**Arrhythmia**	21 (20.8%)	1 (2.2%)	7 (30.4%)	13 (40.6%)	< 0.001
**Tumor history**	16 (15.8%)	6 (13%)	3 (13%)	7 (21.9%)	0.533
**Respiratory diseases**	14 (13.9%)	10 (21.7%)	1 (4.3%)	3 (9.4%)	0.130
**Smoking history**	14 (13.9%)	1 (2.2%)	8 (34.8%)	5 (15.6%)	< 0.001
**History of fragile fracture**	20 (19.8%)	8 (17.4%)	2 (8.7%)	10 (31.3%)	0.101
**Chronic respiratory failure**	5 (5.0%)	4 (8.7%)	0 (0)	1 (3.1%)	0.370
**Chronic cardiac insufficiency**	4 (4.0%)	1 (2.2%)	0 (0)	3 (9.4%)	0.199
**Parkinson’s syndrome**	4 (4%)	2 (4.3%)	0 (0)	2 (6.3%)	0.685
**Long-term oral hormones**	3 (3%)	3 (6.5%)	0 (0)	0 (0)	0.318
**Indwelling urinary catheters**	36 (35.6%)	11 (23.9%)	10 (43.5%)	15 (46.9%)	< 0.001
**Long-term nasal feeding**	35 (34.7%)	7 (15.2%)	9 (39.1%)	19 (59.4%)	0.077

### Laboratory examination and imaging examination

Laboratory examination on the 101 patients after admission showed that peripheral blood leukocytes and percent neutrophils (NEU%) slightly elevated (WBC 10.21 × 10^9^/l ± 4.44 × 10^9^/l, NEU% 78.5 ± 9.98%), and the average hemoglobin (Hgb) was 114.3 ± 20.0 g/l. The inflammatory index showed that C-reactive protein (CRP) was 61.76 ± 62.1 mg/l, procalcitonin (PCT) was 2.07 ± 7.5 ng/ml. HbA1C% was 5.9 ± 0.83%, albumin (ALB) was 37.89 g/l ± 4.88 g/l, while sCr was 69.5 ± 29.56 μmol/l. Blood gas analysis showed that PO_2_ was 76.21 ± 15.2 mmHg and PCO_2_ was 33.48 ± 5.8 mmHg. Chest imaging examination revealed that 65 patients (64.4%) had bilateral lung involvement. Correlation analysis showed that the longer bedridden time was correlated with lower ALB (*P* = 0.006) (Table 2).

**Table 2:** Comparison of first laboratory examinations of elderly bedridden patients with hip fracture and lower respiratory tract infection after admission

Laboratory inspection items	Total	Short-term bedridden group	Middle-term bedridden group	Long-term bedridden group	***P***
**M ± SD**	*N* = 101	*N* = 46	*N* = 23	*N* = 32	
**WBC (× 10**^**9**^**/l)**	10.21 ± 4.44	10.33 ± 3.83	9.6 ± 3.69	10.49 ± 5.67	0.750
**NEU (%)**	78.5 ± 9.98	79.91 ± 11.38	77.84 ± 5.98	77.04 ± 10.13	0.432
**HGB (g/l)**	114.3 ± 20.0	114 ± 19.31	114.2 ± 23.2	114.9 ± 19.1	0.982
**PO**_**2**_ **(mmHg)**	76.21 ± 15.2	74.81 ± 11.25	74.7 ± 12.98	79.1 ± 20.55	0.438
**PCO**_**2**_ **(mmHg)**	33.48 ± 5.8	32.4 ± 4.3	34.3 ± 5.59	34.6 ± 7.52	0.213
**CRP (mg/l),** ***n*** **= 87**	61.76 ± 62.1	80.49 ± 79.12	47.07 ± 38.35	52.56 ± 51.26	0.084
**< 20**	27 (31%)	8 (24.2%)	8 (36.4%)	11 (34.4%)	0.259
**20–120**	50 (57.5%)	18 (54.5%)	13 (59.1%)	19 (59.4%)	
**> 120**	10 (11.5%)	7 (21.2%)	1 (4.5%)	2 (6.3%)	
**PCT (ng/ml),** ***n*** **= 49**	2.07 ± 7.5	0.22 ± 0.38	6.21 ± 13.25	1.25 ± 5.49	0.105
**< 0.1**	28 (57.1%)	9 (64.3%)	5 (45.5%)	14 (58.3%)	0.545
**0.1–25**	18 (36.7%)	5 (35.7%)	4 (36.4%)	9 (37.5%)	
**> 25**	3 (6.1%)	0 (0%)	2 (18.2%)	1 (4.2%)	
**HBA1C%**	5.9 ± 0.83	6.07 ± 0.98	6.02 ± 0.72	5.67 ± 0.59	0.124
**ALB (g/l)**	37.89 ± 4.88	39.56 ± 4.56	36.6 ± 4.39	36.4 ± 5.01	0.006
**sCr (μmol/l)**	69.5 ± 29.56	66.43 ± 22.98	74.4 ± 35.68	70.38 ± 33.4	0.564
**Chest imaging abnormalities**					
**Unilateral**	36 (35.6%)	13 (28.3%)	10 (43.5%)	13 (40.6%)	0.358
**Biolateral**	65 (64.4%)	33 (71.7%)	13 (56.5%)	19 (59.4%)	

### Pathogens

Among the patients, 74 patients (73.3%) received sputum bacteriological culture within 72 h of admission. The positivity rate was 90.5% (67/74 patients), in which 24 (32.4%) had more than two types of bacterial infections. The rate of bacterial infection combined with fungal infection was 66.2% (49 patients).

There were 54 cases found infected with Gram-negative (G^−^) bacilli and 15 cases infected with Gram-positive (G^+^) cocci. Additionally, 2 cases with G^−^ cocci, 49 cases with Candida, and one with *Aspergillus fumigatus* were also detected. The most common pathogens were *Candida albicans* (35 strains), *Klebsiella pneumoniae* (17 strains), *Pseudomonas aeruginosa* (12 strains), and *Candida glabrata* (11 strains). The resistance rate of the bacterial strains was 23.9% (16/67 strains). The resistant bacteria with their rates of resistance included: G^−^ bacilli, such as *Acinetobacter baumannii* (42.9%, 3/7 strains), *Pseudomonas aeruginosa* (25%, 3/12 strains), and *Stenotrophomonas maltophilia* (22.2%, 2/9 strains), and G^+^ cocci, such as *Staphylococcus aureus* (75%, 3/4 strains), *Staphylococcus epidermidis* (33.3%, 1/3 strains) and *Staphylococcus haemolyticus* (20%, 1/5 strains). The resistance rate of the fungi was 2.9% (2/67 strains), and both strains were *Candida krusei* species (Table 3).

**Table 3:** Pathogenic bacterial characteristics of elderly bedridden patients with hip fracture and lower respiratory tract infection

Types	Total (%), ***N*** = 101	Short-term bedridden group (%), ***N*** = 46	Middle-term bedridden group (%), ***N*** = 23	Long-term bedridden group (%), ***N*** = 32	***P***
**Sputum specimen delivery rate**	74 (73.27%)	21 (45.65%)	22 (95.65%)	31 (96.88%)	< 0.001
**Number of positive sputum culture cases,** ***N*** **= 74**	67 (90.54%)	21 (100%)	18 (81.81%)	28 (90.32%)	0.456
**Number of bacterial positive cases**	62 (83.78%)	17 (81.0%)	17 (77.3%)	28 (90.3%)	0.374
**Number of fungal positive cases**	50 (67.57%)	10 (47.6%)	14 (63.64%)	26 (83.87%)	0.022
**Bacterial species (> 2 species)**	24 (32.43%)	10 (47.6%)	7 (31.82%)	7 (22.58%)	0.166
**Bacteria combined with fungi**	49 (66.22%)	9 (42.86%)	14 (63.64%)	26 (83.87%)	0.009
**Number of G**^**−**^ **bacilli cases,** ***N*** **= 67**	54 (80%)	19 (90%)	14 (80%)	21 (76%)	0.813
***Klebsiella pneumonia*** **strains**	17 (25.37%)	2 (9.52%)	8 (44.44%)	7 (25%)	0.042
**Drug-resistant bacteria**	1	0	1	0	-
**Other enterobacteriaceae strains**	17 (25.37%)	6 (28.57%)	4 (22.22%)	7 (25%)	0.939
**Drug-resistant bacteria**	2	1	0	1	-
***Pseudomonas aeruginosa*** **strains**	12 (17.91%)	1 (4.76%)	2 (11.11%)	9 (32.14%)	0.045
**Drug-resistant bacteria**	3	1	1	1	-
***Stenotrophomonas maltophilia*** **strains**	9 (13.43%)	2 (9.52%)	1 (5.56%)	6 (21.43%)	0.319
**Drug-resistant bacteria**	2	0	0	2	-
***Acinetobacter baumannii*** **strains**	7 (10.45%)	2 (9.52%)	3 (16.67%)	2 (7.14%)	0.536
**Drug-resistant bacteria**	3	1	1	1	-
**Other Acinetobacter**	2 (2.99%)	1 (4.76%)	1 (5.56%)	0 (0)	0.506
**Number of G**^**+**^ **coccus cases,** ***N*** **= 67**	15 (22.39%)	6 (28.57%)	5 (27.78%)	4 (14.29%)	0.404
***Staphylococcus aureus*** **strains**	4 (5.94%)	1 (4.76%)	2 (11.11%)	1 (3.57%)	0.675
**Drug-resistant bacteria**	3	0	2	1	-
***Staphylococcus haemolyticus*** **strains**	5 (7.46%)	2 (9.52%)	1 (5.56%)	2 (7.14%)	1.000
**Drug-resistant bacteria**	1	0	1	0	-
***Staphylococcus epidermidis*** **strains**	3 (2.97%)	3 (6.52%)	0 (0)	0 (0)	0.318
**Drug-resistant bacteria**	1	1	0	0	-
**Other strains**	3 (4.48%)	0 (0)	2 (11.11%)	1 (3.57%)	0.349
**Drug-resistant bacteria**	2	0	1	1	-
**Number of G**^**−**^ **coccus (*Moraxella catarrhalis*) cases,** ***N*** **= 67**	2 (2.99%)	0 (0%)	2 (11.1%)	0 (0)	0.069
**Number of enzyme-resistant bacteria**	16 (23.88%)	6 (28.57%)	3 (16.67%)	7 (25%)	0.789
**Number of fungal cases,** ***N*** **= 67**	50 (74.63%)	11 (52.4%)	17 (94.4%)	22 (78.6%)	0.009
***Candida albicans*** **strains**	35 (52.24%)	11 (52.4%)	12 (66.7%)	12 (42.9%)	0.288
***Candida glabrata*** **strains**	11 (16.42%)	2 (9.5%)	3 (16.7%)	6 (21.4%)	0.645
***Candida tropicalis*** **strains**	8 (11.92%)	0 (0)	2 (11.11%)	6 (21.43%)	0.049
***Candida krusei*** **strains**	6 (8.96%)	0 (0)	5 (27.8%)	1 (3.6%)	0.005
***Candida rotundifolia*** **strains**	1 (1.49%)	0 (0)	1 (5.6%)	0 (0)	0.269
***Aspergillus fumigates*** **strains**	1 (1.49%)	0 (0)	0 (0)	1 (3.6%)	1.000
**Number of enzyme-resistant fungi**	2 (2.99%)	0 (0)		2 (7.14%)	-

Correlation analysis showed that the longer bedridden time was positively correlated with higher fungal infections and mixed infections as well as more diversified pathogenic strains (*Pseudomonas aeruginosa*, *Candida tropicalis*). Multidrug-resistant bacteria (MDR) and extensively drug-resistant bacteria (XDR) were also detected in patients with a long-term bedridden period, as shown in Table 3.

### Anti-infection and related therapy

The first choice of antibiotic selection in the 101 patients was cephalosporins (second to fourth generation, 64 patients, 63.4%), carbapenems (31 patients, 30.7%), respiratory quinolones (7 patients, 7%), glycopeptides (4 patients, 4%), and clindamycin (1 patient, 1%). Among these, 31.7% (32 patients) needed expansion of their antibiotic coverage. The median time from the first antibiotic use to wider coverage was 6 days (IQR 3.5 days to 7 days). A total of 32 patients (31.7%) received antifungal treatment at the first visit, in which 53.1% (17 patients) required fluconazole and 9.4% (3 patients) needed multiple antifungal drugs. The median time from antifungal treatment to drug coverage expansion was 8.5 days (IQR 5 days to 15.5 days). During treatment, 26 patients (25.7%) received parenteral nutrition support, 12 patients (11.9%) received glucocorticoids, and 9 patients (8.9%) underwent endotracheal intubation or tracheotomy.

Correlation analysis showed that the longer the bedridden time was correlated with the requirement for antifungal treatment, antibiotic coverage expansion, parenteral nutritional support, and the number of antibiotic upgrades (*P* < 0.05) (Table 4).

**Table 4:** Comparison of antibiotics and related treatments in elderly bedridden patients with hip fracture and lower respiratory tract infection

Treatment	Total (%), ***N*** = 101	Short-term bedridden group (%), ***N*** = 46	Middle-term bedridden group (%), ***N*** = 23	Long-term bedridden group (%), ***N*** = 32	***P***
**Initiative antibiotic types**					
**Second-generation cephalosporins**	10 (9.9%)	3 (6.5%)	4 (17.4%)	3 (9.4%)	0.353
**Third-generation cephalosporins (without enzyme inhibitors)**	31 (30.7%)	18 (39.1%)	5 (21.7%)	8 (25.1%)	0.235
**Third-generation cephalosporins (with enzyme inhibitors)**	22 (21.8%)	5 (10.9%)	11 (47.8%)	6 (18.8%)	0.002
**4th-generation cephalosporin**	1 (1%)	0 (0)	0 (0)	1 (3.1%)	0.545
**Carbapenems**	31 (30.7%)	17 (37%)	1 (4.3%)	13 (40.6%)	0.007
**Quinolones**	7 (7%)	2 (4.3%)	3 (13%)	2 (6.3%)	0.469
**Glycopeptides**	4 (4%)	2 (4.3%)	0 (0)	2 (6.3%)	0.685
**Clindamycin**	1 (1%)	1 (2.2%)	0 (0)	0 (0)	1.000
**Antibiotics need to be upgraded**	32 (31.7%)	7 (15.2%)	8 (34.8%)	17 (53.1%)	0.002
**Upgrade M (IQR) on the X-day of admission**	6 (3.5–7)	5 (3–7)	6 (4–10)	6.5 (3.0–7.5)	0.259
**Number of antibiotic upgrades M (IQR)**	0 (0–1)	0 (0–0)	0 (0–1)	1 (0–2)	0.001
**Antifungal therapy**	32 (31.7%)	6 (13.0%)	6 (26.1%)	20 (62.5%)	< 0.001
**Fluconazole is preferred**	17 (16.8%)	3 (6.5%)	5 (21.7%)	9 (28.1%)	0.032
**Upgrading ratio of antifungal drugs**	3 (9.38%)	0 (0)	1 (16.7%)	2 (10%)	1.000
**Upgrade M (IQR) on the X-day of admission**	8.5 (5–15.5)	5.5 (4–13)	7 (7–10)	11.5 (5.5–20)	0.407
**Upgrading times of antifungal agents M (IQR)**	1 (1–1)	-	1	1	-
**Need parenteral nutrition support**	26 (25.7%)	6 (13%)	6 (26.1%)	14 (43.8%)	0.010
**Use of glucocorticoids**	12 (11.9%)	5 (10.9%)	1 (4.3%)	6 (18.8%)	0.255
**Intravenous glucocorticoid**	9 (8.91%)	2 (4.3%)	1 (4.3%)	6 (18.8%)	
**Oral glucocorticoid**	3 (2.97%)	3 (6.5%)	0	0	0.086
**Not used**	89 (88.12%)	41 (89.1%)	22 (95.7%)	26 (81.3%)	
**Tracheal intubation/incision**	9 (8.9%)	2 (4.3%)	1 (4.3%)	6 (18.8%)	0.061

### Univariate analysis and binomial logistic regression analysis related to disease prognosis

All the collected variables were included in the univariate analysis of death-related factors. It was shown that indwelling nasal feeding, serum albumin level, infection with drug-resistant bacteria, antibiotics to be upgraded, numbers of antibiotics upgrade, use of glucocorticoids during antibiotic therapy, use of glucocorticoids, and parenteral nutrition support were correlated with the mortality in elderly bedridden patients with hip fracture and lower respiratory tract infection (*P* < 0.05) (Table 5).

**Table 5:** Univariate analysis of mortality in elderly bedridden patients with hip fracture and lower respiratory tract infection

Number	Variable	OR (95% CI)	***P***
**1**	Age	1.10 (1.01–1.20)	0.025
**2**	Bedridden time after hip fracture	1–12 month, 1.37 (0.21–8.80)	0.003
	Reference, < 1 month	> 12 month, 8.60 (2.18–33.9)	
**3**	Indwelling nasal feeding	9.16 (2.70, 31.1)	< 0.001
**4**	Number of hospitalizations in the past year	1.47 (1.09–1.98)	0.011
**5**	Days of hospitalization	1.04 (1.01–1.08)	0.027
**6**	Serum albumin level	0.79 (0.68–0.90)	< 0.001
**7**	Infection with drug-resistant fungus	7.55 (2.19, 26.1)	0.001
**8**	Infection with drug-resistant bacteria	10.7 (3.22–35.4)	< 0.001
**9**	Third-generation cephalosporins (without enzyme inhibitors)	3.17 (1.09–9.24)	0.034
**10**	Antibiotics need to be upgraded	7.68 (2.41–24.4)	< 0.001
**11**	Numbers of antibiotics upgrade M	2.27 (1.43–3.61)	< 0.001
**12**	Use of antifungal drugs	5.50 (1.81–16.7)	0.003
**13**	Use of glucocorticoids during antibiotic therapy	Oral, 5.06 (0.41–62.1)	< 0.001
	Reference, not used	Intravenous, 81 (8.96–732)	
**14**	Use of glucocorticoids	30.4 (6.81–135)	< 0.001
**15**	Parenteral nutrition support	8.43 (2.70–26.4)	< 0.001
**16**	Tracheal intubation or tracheotomy	4.86 (1.15–20.5)	0.031

These single factors were further incorporated into the binomial logistic regression model. The independent risk factors related to the prognosis of the disease were the use of glucocorticoids during the anti-infective period, the prolongation of bedridden time, and low serum albumin levels. Further stratification of two covariates, the use of glucocorticoids during the anti-infective period and the prolongation of bedridden time, showed that the mortality risk of patients with lower respiratory tract infections who received intravenous glucocorticoid infusion during anti-infective treatment was 89.4 times higher than that of those who did not use glucocorticoids (dummy variable *P* = 0.025).

The mortality risks of the oral glucocorticoids used patients and the non-glucocorticoids used patients were not statistically significantly different. The mortality risk of patients with lower respiratory tract infections who were bedridden for more than 1 year was 10.4 times higher than that of those who were bedridden for less than 1 month (dummy variable *P* = 0.015). There was no significant difference between those who were bedridden for 1 to 12 months and those who were bedridden for less than 1 month. Simultaneously, patients with higher serum albumin levels also showed a lower mortality risk (*P* = 0.020, OR = 0.81) (Table 6).

**Table 6:** Binomial logistic regression of mortality in elderly bedridden patients with hip fracture and lower respiratory tract infection

Variable	OR	95% CI	***P***
**Use of glucocorticoids during antibiotic therapy**			0.006
**Intravenous use**	89.4	5.52–999	0.025
**Oral administration**	12.5	0.41–377	0.868
**Not used**	Reference		
**Bedtime**			0.051
**> 12 month**	10.4	1.05–123	0.015
**1–12 month**	1.5	0.1–26.6	0.514
**< 1 month**	Reference		
**Serum albumin level**	0.81	0.67–0.97	0.020

## Discussion

This is the first study to analyze the etiological characteristics and prognostic risk factors of lower respiratory tract infection in elderly patients with hip fracture during different bedridden periods. The elderly patients with hip fracture who were bedridden for a prolonged period (> 12 months) had the following characteristics, advanced age, repeated hospitalizations, and multiple underlying diseases, such as cerebrovascular diseases, dementia, coronary heart disease, and arrhythmia, in addition to smoking history, long-term catheterization, or nasogastric feeding. Bivariate logistic regression analysis showed that bedridden time > 1 year was a high-risk factor for poor prognosis. Sheikh et al. showed that lower respiratory tract infection was the leading cause of mortality 30 days postoperatively (44.1%, 52/118 cases) and one of the most significant predictors of mortality, which was in accordance with our results [11]. Other studies have reported that the 1-year all-cause mortality rate of these patients after fracture is approximately 8.4–36% [2, 10, 16–18]. The 5-year (3.5–8 years) mortality rate is 74–79% [19, 20]. Friesendorff et al. have conducted a follow-up of more than 20 years and showed that the all-cause mortality rate of elderly hip fracture patients continued to be higher than that of the control group at the end of follow-up [14].

Due to frailty, low immunity, and poor nutritional status, the hemogram, CRP, PCT, and other inflammatory indexes in the elderly are often not positively correlated with lower respiratory tract infection. In this study, WBC, NEU%, and inflammatory markers in the peripheral blood were slightly elevated or even normal. With the prolongation of the time being bedridden, the serum albumin level tended to decrease, and the proportion of patients requiring parenteral nutritional support increased. It indicated that the nutritional status of the patients deteriorated, which affected the prognosis of the disease.

The effect of age on the prognosis of patients with hip fractures remains controversial. Most studies supported that increased age was an independent risk factor for re-admission and death in elderly patients with hip fracture [21–23]. Bohl et al. [23] proposed that age ≥ 90 years is an independent risk factor for postoperative pulmonary infection in elderly patients with hip fracture. The increase in the mortality rate of older patients is thought to be associated with other factors, such as prolonged bedridden time and decreased nutritional status, rather than advanced age itself.

In terms of pathogenic bacteria, our study showed that the infectious pathogens causing the lower respiratory tract infections in the elderly were G^−^ bacilli and fungi, followed by G^+^ cocci. The predominant G^−^ bacilli were *Klebsiella pneumoniae*, *Pseudomonas aeruginosa*, *Stenotrophomonas maltophilia*, and *Acinetobacter baumannii*. These strains were resistant to various degrees and included MDR *Pseudomonas aeruginosa* resistant to carbapenem in one patient, XDR *Acinetobacter baumannii* in two patients, and levofloxacin resistant *Stenotrophomonas maltophilia* in two patients, of which one strain was also resistant to minocycline, which made anti-infective treatment difficult. The rate of fungal infection in this study was significantly higher in previous studies [24, 25]. The difference was possibly caused by acute stress status, dysbiosis, or displacement of oral flora. There were 15 patients with G^+^ cocci infection and nearly half comprised drug-resistant strains (46.7%), among which the most common was *Staphylococcus aureus* at 75% (3/4). This proportion of MRSA was higher than that reported in Beijing (36.3% in 2017) [26]. However, our results were consistent with the infection and drug-resistant proportion in elderly patients with pulmonary infection published by the National Bacterial Resistance Gene Network [24].

When bedridden time is prolonged, the pathogens of the lower respiratory changes with following characteristics: (1) There is an increase in bacterial species, mainly the G^−^ bacilli, and fungal species. (2) An increase in multidrug-resistant and extensively drug-resistant G^−^ bacilli is observed. (3) There is an increase in the proportion of bacteria to fungal species. (4) An increase in *Candida tropicalis* and *Candida krusei* develops among the fungi. There might be several reasons for these changes. First, at the early stage following fracture, the lung infection is mostly mild. There is no chronic debilitating status, body resistance is normal, and the proportion of opportunistic infectious pathogens is relatively small. Second, with the prolongation of the bedridden period, the patient’s swallowing reflex function is reduced. Aspiration and choking increase the proportion of oral bacteria, anaerobic bacteria, and fungi-induced lung infections. The proportion of pathogenic bacteria increase, the sputum becomes more viscous, and infection is not as easy to control or prevent. In addition, the unreasonable or repeated use of antibiotics and increased hospitalizations raise the risk of drug-resistant bacterial infection.

With regard to the application of antibiotics, we found that with the prolongation of bedridden time, the efficacy of the first empirical antibiotic treatment gradually decreased, while the proportion of antibiotic adjustment or required expansion of coverage increased. Due to conventional sputum culture, the average antibiotic adjustment time was 6 days after admission (IQR 3.5 to 7 days), and it may have even delayed the recovery from illness. Therefore, the clinician should try to use pathogen genetic detection to assist the first reasonable choice of antibiotics.

Oral glucocorticoids or intravenous infusion of glucocorticoids was associated with poor prognosis of these patients. There were several reasons for this result. First, the risk of opportunistic infections would increase. As glucocorticoids have a strong inhibitory effect on cellular and humoral immunity, they are likely to increase the risk of pathogenic infection and blood transmission. Second, metabolic abnormalities are caused by glucocorticoids, such as elevated blood glucose, increased infection risk, and electrolyte disorders. Third, mental disability in the elderly can present as insomnia, euphoria, depression, delusions, or hallucinations, or with other atypical symptoms. Fourth, increased blood volume with the retention of sodium and water can lead to congestive heart failure, intracranial hypertension, and high blood pressure. Finally, glucocorticoids can cause gastrointestinal abnormalities, such as peptic ulcer and pancreatitis. Therefore, glucocorticoids should be used with caution in elderly patients with hip fracture and pulmonary infection, particularly in those suspected of having opportunistic infections or bacteremia.

There are several limitations in this study. The sample size is small. There was no quantitative assessment of each patient’s condition, and the follow-up time was relatively short. Further study with a larger sample size and prospective design was still needed.

## Conclusions

In conclusion, with the prolongation of bedridden time in elderly patients with hip fracture, physiological reflexes showed functional deterioration in areas such as swallowing, coughing, and urination. The efficacy of empiric antibiotic therapy is decreased, and mortality is increased. Elderly hip fracture patients with prolonged bedridden time had an increased chance of opportunistic pulmonary infection and decreased nutritional status. Glucocorticoids should be used cautiously.

## Supplementary Information

**Additional file 1 **: **Supplement Table 1**. Comparison of basic information of elderly bedridden patients with hip fracture and lower respiratory tract infection

## Data Availability

The datasets used or analyzed during the current study are available from the corresponding author on reasonable request.

## Abbreviations

CRP: C-reactive protein
PCT: Procalcitonin
HAP: Hospital-acquired pneumonia
Hgb: Hemoglobin
ALB: Albumin
MDR: Multidrug-resistant bacteria
XDR: Extensively drug-resistant bacteria
