# Supplementary material for: Elderly patients with concurrent hip fracture and lower respiratory tract infection: the pathogens and prognosis over different bedridden periods
Source: J Orthop Surg Res. 2021 Apr 13;16:246. doi: 10.1186/s13018-021-02399-1 (PMC8042877; doi:10.1186/s13018-021-02399-1)
Supplement: Supplementary file 1 — Additional file 1 : Supplement Table 1. Comparison of basic information of elderly bedridden patients with hip fracture and lower respiratory tract infection [file 13018_2021_2399_MOESM1_ESM.docx]

**Supplement Table 1. Comparison of basic information of elderly bedridden patients with hip fracture and lower respiratory tract infection**

| **Basic information** | | **Total** | **Short-term**  **bedridden group** | **Middle-term bedridden group** | **Long-term**  **bedridden group** | **P** |
| --- | --- | --- | --- | --- | --- | --- |
| **/ (M±SD)** | | N=101 | n=46 | n=23 | n=32 |  |
| **Gender male** | | 46(45.5%) | 18(39%) | 12(52%) | 16(50%) | 0.490 |
| **Age M ± SD (yrs)** | | 82.31±7.98 | 81.83±7.42 | 79.7±9.19 | 84.87±7.32 | 0.049 |
| **> 80 yrs** | | 64(63.4%) | 26(56.5%) | 12(52.2%) | 26(81.3%) | 0.037 |
| **Fracture types** | Fracture of femoral neck  Intertrochanteric fracture | 64(63.4%) | 27(58.7%) | 14(60.9%) | 23(71.9%) | 0.474 |
|  |  | 37(36.6%) | 19(41.3%) | 9(39.1%) | 9(28.1%) |  |
| **Treatment of fracture** | Joint replacement  Internal fixation    Conservative treatment | 48(47.5%) | 18(39.1%) | 13(56.5%) | 17(53.1%) | 0.625 |
|  |  | 29(28.7%) | 16(34.8%) | 5(8.7%) | 8(25.0%) |  |
|  |  | 24(23.8%) | 12(26.1%) | 5(8.7%) | 7(21.9%) |  |
| **Bed time (month)** | | 17.45±31.31 | 0.28±0.51 | 4.18±1.8 | 51.69±37.17 | <0.001 |
| **Number of hospitalizations in the past year**  **M (IQR)** | | 0(0-1) | 0(0-0) | 0(0-1) | 1(0-3) | <0.001 |
| **The number of days in hospital** | | 17.9±13.4 | 11.2±11.01 | 22.87±11.96 | 23.97±13.46 | <0.001 |
| **Number of deaths** | | 17(16.83%) | 3(6.52%) | 2(8.70%) | 12(37.5%) | <0.001 |
| **Direct death due to severe pneumonia** | | 16(94.12%) | 2(66.67%)^*^ | 2(100%) | 12(100%) | 0.294 |

* Another patient died because of advanced pancreatic cancer.
